# Phenotypic Switch Induced by Simulated Microgravity on MDA-MB-231 Breast Cancer Cells

**DOI:** 10.1155/2014/652434

**Published:** 2014-08-18

**Authors:** Maria Grazia Masiello, Alessandra Cucina, Sara Proietti, Alessandro Palombo, Pierpaolo Coluccia, Fabrizio D'Anselmi, Simona Dinicola, Alessia Pasqualato, Veronica Morini, Mariano Bizzarri

**Affiliations:** ^1^Department of Clinical and Molecular Medicine, “Sapienza” University of Rome, Piazza Sassari 3, 00161 Rome, Italy; ^2^Department of Surgery “PietroValdoni”, “Sapienza” University of Rome, Via A. Scarpa 14, 00161 Rome, Italy; ^3^Department of Experimental Medicine, “Sapienza” University of Rome, Systems Biology Group, Viale Regina Elena 324, Via A. Scarpa 14, 00161 Rome, Italy

## Abstract

Microgravity exerts dramatic effects on cell morphology and functions, by disrupting cytoskeleton and adhesion structures, as well as by interfering with biochemical pathways and gene expression. Impairment of cells behavior has both practical and theoretical significance, given that investigations of mechanisms involved in microgravity-mediated effects may shed light on how biophysical constraints cooperate in shaping complex living systems. By exposing breast cancer MDA-MB-231 cells to simulated microgravity (~0.001 g), we observed the emergence of two morphological phenotypes, characterized by distinct membrane fractal values, surface area, and roundness. Moreover, the two phenotypes display different aggregation profiles and adherent behavior on the substrate. These morphological differences are mirrored by the concomitant dramatic functional changes in cell processes (proliferation and apoptosis) and signaling pathways (ERK, AKT, and Survivin). Furthermore, cytoskeleton undergoes a dramatic reorganization, eventually leading to a very different configuration between the two populations. These findings could be considered adaptive and reversible features, given that, by culturing microgravity-exposed cells into a normal gravity field, cells are enabled to recover their original phenotype. Overall these data outline the fundamental role gravity plays in shaping form and function in living systems.

## 1. Introduction

Space flights induce relevant changes in human physiology, such as bone loss, muscle atrophy, deregulation of immune function, hematological anomalies, and cardiovascular function impairment. Microgravity effects may be ascribed to systemic interferences with body fluids distribution, disappearance of fluid shear, perturbation of the circadian clock, altered endothelial function, and reduced loading on skeletal structures [1]. Yet, a direct effect on cell and signaling pathways inside the cell has been documented, despite the fact that microgravity has been previously thought to be too weak for contrasting the intermolecular forces [2]. Thereby, it is likely that spaceflight could exert its detrimental effects on astronauts via changes in cellular structure and/or functions.

Several studies, performed both in simulated and actual microgravity, have shown that normal as well as neoplastic cells undergo dramatic changes after exposition to a microgravity field. Cell morphology, as well as features of subcellular organelles and cytoskeleton structure, has been reported to be dramatically influenced by gravity [3, 4]. Similarly, relevant modifications in tissue organization have been recorded in microgravity-exposed organs and/or animals [5, 6]. Shape changes are likely to be mediated by concomitant structural rearrangement of cytoskeleton (CSK), which is severely disorganized under microgravity [7, 8]. CSK conveys mechanical signals into the cells, and by that way it influences both biochemical pathways [9, 10] and gene expression [11, 12]. As a consequence, many metabolic, proliferative, and differentiating processes end up to be deeply perturbed [13].

Microgravity effects may be ascribed to both indirect and direct effects [14]. Meanwhile specialized cells and structures in the plant realm have been found to be sensitive to even subtle change in gravity vector [15]; no components in the mammalian cells have been so far identified as having a sufficiently large mass density difference in respect to the surrounding medium: thus, the force exerted by the gravitational field is nowhere higher than the energy of random thermal motion and cannot significantly modify the behaviour of any single subcellular structure. Instead, mammalian cells may be able to sense some environmental changes due to gravity affecting a wide range of biophysical parameters: buoyancy, shear forces, viscosity, diffusion process, and many others. Yet, a lot of gravity-related phenomena at the cellular level, involving shape rearrangement, cytoskeleton disruption, and even modified gene expression, would hardly be explained by only considering changes in “external” environmental biophysical parameters. Indeed, gravity may likely affect some general properties of the systems, acting “directly” as an organizing field parameter. We have previously reported that by “removing” the gravitational constraint, according to the nonequilibrium theory [16], murine osteoblasts underwent a transition after a bifurcation point, thus recovering degrees of freedom enabling the system in accessing new attractor states, that is, new phenotypic configurations [17]. Indeed, microgravity induces the emergence of two distinct phenotypes, characterized by different morphologies. Herein we investigate if a similar pattern could be retrieved in breast cancer cells and how such features are associated with differences in their biochemical pathways. Indeed, conflicting data have been reported by investigations carried out on cancer cells exposed to microgravity: some authors have recorded an overall inhibitory effect on cancer cell proliferation, motility, and survival [18, 19], whereas others have observed the opposite [20–22]. We hypothesize that such results may be likely explained by the emergence of distinct cell phenotypes, characterized by different functional and reproductive features.

## 2. Material and Methods

### 2.1. RPM (Random Positioning Machine)

Microgravity conditions were simulated by a Desktop RPM, a particular kind of 3D clinostat [23], manufactured by Dutch Space (Leiden, The Netherlands). The degree of microgravity simulation depends on angular speed and on the inclination of the disk. These tools do not actually eliminate the gravity but allow you to apply a stimulus rather than a unidirectional omnidirectional 1 g. Effects generated by the RPM are comparable to those of the real microgravity, provided that the direction changes are faster than the response time of the system to gravity field. The desktop RPM was located in a standard incubator (to maintain temperature, CO_2_, and humidity levels) and connected to the control console through standard electric cables.

### 2.2. Cell Culture

MDA-MB-231 human breast cancer cell line was purchased from European Collection of Cell Cultures (ECACC, Sigma-Aldrich, St. Louis, MO, USA). Cells were seeded into Nunc OptiCell Cell Culture Systems, gas-permeable cell culture disks (Thermo Scientific, Rochester, USA), and cultured in Dulbecco's modified Eagle's medium (DMEM, Euroclone Ltd., Cramlington, UK) supplemented with 10% Fetal Bovine Serum (FBS, HyClone Laboratories, Logan, UT, USA), 200 mM L-glutamine, 100 IU/mL Penicillin, and 100 *μ*g/mL Streptomycin (all from Euroclone Ltd., Cramlington, UK). Then OptiCells containing subconfluent monolayers were fixed onto the RPM, as close as possible to the center of the platform, which was rotated at a speed of 60°/s using the random mode of the machine. On ground control (1 g static cultures) and RPM cultures were kept in the same humidified incubator at 37°C in an atmosphere of 5% CO_2_ in air. Experiments were performed for 24 and 72 hours. After 24 and 72 hours of microgravity exposure, cell clumps swimming in culture supernatants were found, in addition to adherent cells, and separately collected. The three cell populations (on ground control cells, RPM adherent cells, and RPM cell clumps) were characterized separately.

### 2.3. Optical Microscopy

Cell clumps were collected, washed in PBS, and deposited onto a clearly defined area of a glass slide using a Shandon CytoSpin 4 Cytocentrifuge, Thermo Scientific, while maintaining cellular integrity. Cell clumps and adherent and on ground control cells were fixed in 4% paraformaldehyde for 10 minutes at 4°C and photographed with Nikon Coolpix 995 digital camera coupled with Zeiss Axiovert optical microscope. The images were obtained with a 320x magnification, saved as TIFF files, and used for image analysis.

### 2.4. Image Analysis

Image analysis was performed on 10 images for each group of MDA-MB-231 cells. As the analysis was performed blindly, the image groups were classified as follows: A (on ground cells, 24 h), B (RPM adherent cells, 24 h), C (RPM cell clumps, 24 h), D (on ground cells, 72 h), E (RPM adherent cells, 72 h), and F (RPM cell clumps, 72 h). In each image, single randomly chosen cells (50 for each group) were contoured with a fine black marker by different researchers, simply scanned, and cataloged according to the time of study: 24 and 72 hours. This method was chosen because pathologists are used to correlate the shape the cells acquire with their malignancy by means of morphological, qualitative, and subjective observations. Thus, we decided to perform a semiautomatic analysis, coupling the expertise of researchers with a computerized parameterization method. All the images were processed by Adobe Photoshop CS4. All the pictures (i.e., all the sheets of the groups, for each time point) were resized at 2560 × 1920 pixels according to original scale of image acquisition. For each black contoured cell, edges were refined. Then cells were black filled and threshold was adjusted in order to exclude from the image other cells and background. For each time point a single sheet of all the cells considered was created. To obtain single cell shape parameters (area *A*, roundness, solidity, and fractal dimension FD), ImageJ v1.47h software was used. Then, the software analyzed single cells, by the function “shape descriptor.” In addition to area *A* were calculated

(1) $$\begin{array}{c} \text{Roundness}=\frac{4A}{\pi \sqrt{\text{ma}}}\\ \text{Solidity}=\frac{A}{\text{CA}}, \end{array}$$

where *A* is the area of the cell, ma is the major axis, and CA is the convex area, namely, the area of the convex hull of the region. The convex hull of a region is the smallest region that satisfies two conditions: (a) it is convex and (b) it contains the original region.

As for FD, it was obtained by means of box counting method using FracLac plugin:

(2) $$\begin{array}{c} \text{FD}=\underset{\varepsilon \to 0}{\lim}\left[1-\frac{\log\left[L_{\varepsilon }\left(C\right)\right]}{\log\varepsilon }\right], \end{array}$$

where *C* is the considered curve, *L* is the length of the curve *C*, and *ε* is the length of the segment used as unit to calculate *L*.

Single graphs about roundness, solidity, and FD were obtained for each set of images.

### 2.5. Fluorescence Microscopy

MDA-MB-231 cells were fixed in 4% paraformaldehyde for 10 minutes at 4°C and incubated over night at 4°C with PBS (CMF, Calcium, and Magnesium Free) 1,5% goat serum plus the following specific antibodies: anti-*α*-tubulin (T5168, Sigma-Aldrich) and anti-vimentin (sc-6260, Santa Cruz biotechnology). For F-actin visualization rhodamine-phalloidin (Invitrogen Molecular Probes, Eugene) was used. Cells were washed three times with PBS (1% BSA 0.2% Triton X 100) and incubated with rhodamine-phalloidin, the anti-mouse IgG-FITC PN IM1619 secondary antibody (Beckman-Coulter Inc., Fullerton, CA, USA), and HOECHST 33342 (Sigma-Aldrich, St. Louis, MO, USA) to stain the DNA. Finally, cells were washed, mounted in buffered glycerol (0.1 M, pH 9.5), and analyzed using a Zeiss Fluorescent Microscope. The images were scanned under 40x objective.

### 2.6. Cell Cycle Analysis

Cell clumps were collected and centrifuged and pellets were trypsinized and washed twice with PBS (Phosphate Buffered Saline, Sigma-Aldrich, St. Louis, MO, USA). Adherent and ground control cells were trypsinized and washed twice with PBS. Cells were fixed with 70% ethanol at 4°C for 24 h and stained with DNA PREP Stain (Beckman Coulter, Fullerton, USA) at 4°C overnight. Stained cells were measured by flow cytometry. Cell cycle analysis was performed three times.

### 2.7. Annexin V/7-AAD Staining

Cell clumps were collected and centrifuged and pellets were trypsinized and washed twice with PBS. Adherent cells and ground control cells were trypsinized and washed twice with PBS. The cells were stained with FITC labeled annexin V/7-AAD (7-aminoactinomycin-D) according to the manufacturer's instructions (annexin V/7-AAD kit; Beckman Coulter, Marseille, France). Briefly, a washed cell pellet (5 × 10^4^ cells/mL) was resuspended in 500 *μ*L binding buffer; 10 *μ*L of annexin V together with 20 *μ*L 7-AAD was added to 470 *μ*L cell suspension. The cells were incubated for 15 min on ice in the dark. The samples were analyzed by flow cytometry. Apoptosis assay was performed three times.

### 2.8. Flow Cytometry

Flow cytometry was performed using an EPICS Coulter XL (Beckman-Coulter Inc.). The fluorescence of 20,000 events was measured. An excitation wavelength of 488 nm was used in combination with standard filters to discriminate between the FL1 (forward scatter) and FL3 (side scatter) channels. Data were analyzed by ModFit LT Software (Verity Software Inc., USA).

### 2.9. Western Blot

Cell clumps were washed twice with ice-cold PBS and resuspended in RIPA lysis buffer (Sigma Chemical Co.). Adherent and ground control cells were washed twice with ice-cold PBS and scraped in RIPA lysis buffer (Sigma Chemical Co.). A mix of protease inhibitors (Complete-Mini Protease Inhibitor Cocktail Tablets, Roche, Mannheim, Germany) and phosphatase inhibitors (PhosStop; Roche, Mannheim, Germany) was added just before use. Cellular extracts were then centrifuged at 8,000 ×g for 10 min. The protein content of supernatants was determined using the Bradford assay. For western blot analysis, cellular extracts were separated on SDS-polyacrylamide gels with a concentration of acrylamide specific for the proteins studied. Proteins were blotted onto nitrocellulose membranes (BIO-RAD, Bio-Rad Laboratories, Hercules, CA, USA) and probed with the following antibodies: anti-Cyclin D1 (AB-90009) from Immunological Sciences; anti-survivin (number 2808), anti-phospho-AKT (ser473) (number 9271S), anti-AKT (number 9272S), anti-phospho-ERK1/2 (number 9106), anti-cleaved PARP (number 9541), anti-GAPDH (number 2118), all from Cell Signaling Technology; anti-Bax (sc-493), anti-Bcl-2 (sc-492), anti-ERK1 (sc-94), all from Santa Cruz Biotechnology. Antigens were detected with enhanced chemiluminescence kit (Amersham Biosciences, Little Chalfont Buckinghamshire, England), according to the manufacturer's instructions. All Western blot images were acquired and analyzed through Imaging Fluor S densitometer (Biorad-Hercules).

### 2.10. Statistical Analysis

Data were expressed as mean ± standard deviation (SD) or mean ± standard error (SE). Data were statistically analyzed with the analysis of variance (ANOVA) followed by the Bonferroni post-test. Differences were considered significant at the level of *P* < 0,05. Statistical analysis was performed by using GraphPad Instat software (GraphPad Software, Inc.; San Diego, CA, USA).

## 3. Results 

### 3.1. Effect of Microgravity on MDA-MB-231 Morphology

**Figure 1:**
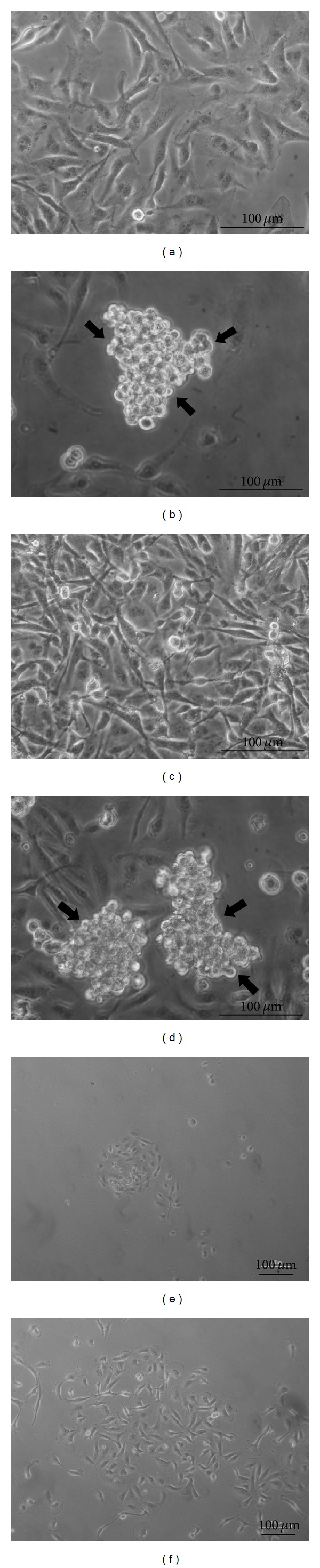
Microphotographs of MDA-MB-231 by optical microscopy. MDA-MB-231 cell line in on ground control condition at 24 (a) and 72 hours (c). MDA-MB-231 cells exposed to microgravity for 24 (b) and 72 hours (d). RPM cell clumps, after reseeding into a normal gravitational field after 6 (e) and 24 hours (f). Magnification ×320 (a), (b), (c), (d), ×100 (e), (f).

MDA-MB-231 cell line grew as a monolayer when cultured under static 1 g condition (on ground control, Figures 1(a) and 1(c)). After 24 and 72 hours of simulated microgravity exposure, cells were distributed into two populations: the first, adhering to the substrate, represented by flat, spindle cells; the second, represented by rounded cells, aggregated in cell clumps, floating in the culture medium (Figures 1(b) and 1(d)). This distribution does not represent a transitory state, given that the percentage of cells at both 24 and 72 hours still remains constant. However, beside the fact that such changes are likely to involve several modifications on shape and biological function, the observed nonadherent phenotype is still wholly reversible after 72 hours. Indeed, after reseeding into a normal gravitational field, cell clumps were* de novo* able to adhere to the culture plate already after 6 hours (Figure 1(e)) and to fully recover their native morphological traits and topological distribution after 24 hours (Figure 1(f)).

### 3.2. Effect of Microgravity on Quantitative Morphological Parameters

Quantitative image analysis was performed by quantifying roundness, solidity, and fractal dimension (FD). Significant differences were recorded among the three cell populations (Table 1).* Roundness*: no statistically significant differences between on ground cells and RPM adherent cells have been observed at both 24 and 72 hours. Instead, RPM cell clumps showed a significant strong increase in roundness compared to control and RPM adherent cells at 24 and 72 hours.* Solidity*: at 24 hours, no statistically significant differences between on ground cells and RPM adherent cells were recorded; meanwhile in RPM cell clumps the solidity index was significantly higher with respect to on ground cells and RPM adherent cells. At 72 hours, the solidity index significantly increased in both RPM cell populations, reaching its highest level in RPM cell clumps.* Fractal dimension*: no statistically significant differences between on ground cells and RPM adherent cells were recorded both at 24 and 72 hours. Instead, RPM cell clumps showed a statistically significant decrease in FD compared to control and RPM adherent cells at 24 and 72 hours. These results are coherent with the qualitative morphological assessment and confirmed that microgravity exposure leads to the emergence of two morphologically distinct cell populations.

### 3.3. Effect of Microgravity on MDA-MB-231 Cytoskeleton Architecture

**Figure 2:**
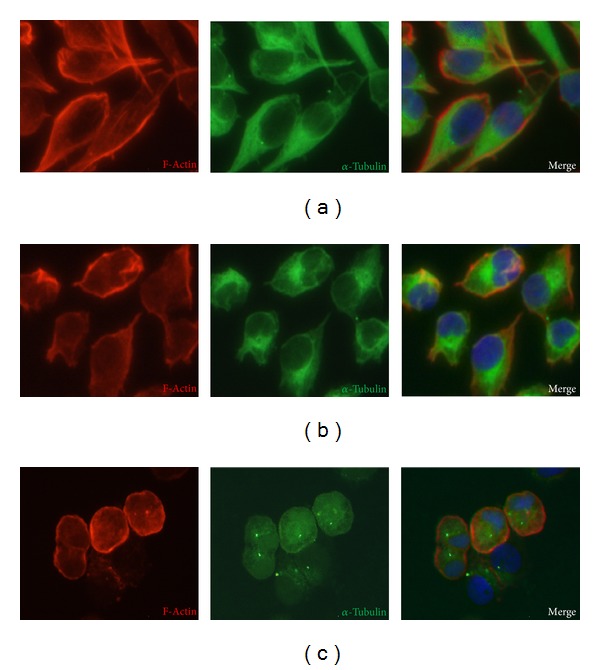
Immunofluorescence images of F-actin and *α*-tubulin in MDA-MB-231. Rhodamine-phalloidin staining of MDA-MB-231 showing F-actin distribution patterns (red color) and immunostaining of *α*-tubulin (green color) and HOECHST 33342 to stain nuclei (blue color) after 24 hours in on ground control cells (a), RPM adherent cells (b), and RPM cell clumps (c). Magnification ×400.

**Figure 3:**
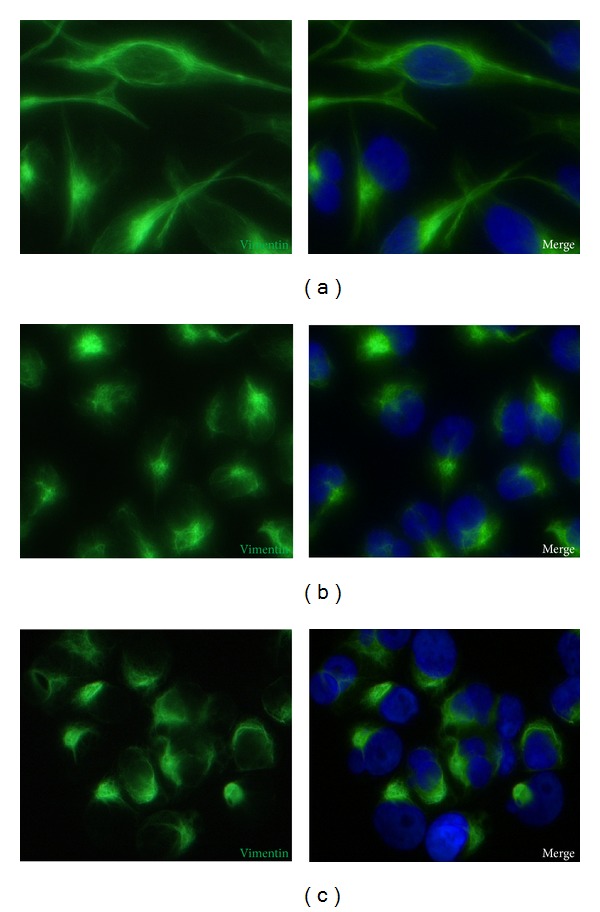
Immunofluorescence images of vimentin in MDA-MB-231. Immunostaining of vimentin (green color) and HOECHST 33342 to stain nuclei (blue color) after 24 hours in on ground control cells (a), RPM adherent cells (b), and RPM cell clumps (c). Magnification ×400.

After 24 hours of microgravity exposure, both MDA-MB-231 RPM adherent cells and RPM cell clumps showed a large rearrangement of F-actin, *α*-tubulin, and vimentin compared to on ground control cells. In on ground control cells the network of cytosolic F-actin bundles appeared well organized and the microtubules appeared orderly radiating from the perinuclear area throughout the cytoplasm toward the cell periphery (Figure 2(a)). In RPM adherent cells the actin filaments showed a disappearance of the complex cytosolic network which appeared mostly localized on the cell border; microtubules were disorganized, with a more evident thickening in perinuclear position (Figure 2(b)). In floating cell clumps, the actin meshwork appeared completely disrupted, and the filaments were mainly localized behind the cell border. Tubulin meshwork was also completely disrupted and a slight diffuse fluorescence was observed spreading throughout the entire cytoplasm (Figure 2(c)). In the on ground cells vimentin filaments were well organized all over the cytoplasm (Figure 3(a)). In both RPM adherent cells and cell clumps the vimentin network was disrupted, appearing in the form of dense aggregates closely associated with the nucleus (Figures 3(b) and 3(c)). Cytoskeleton rearrangements were almost stable, given that no significant changes have been observed even after 72 hours in microgravity-exposed cells (data not shown).

### 3.4. Microgravity Modifies MDA-MB-231 Cell Cycle Distribution

**Figure 4:**
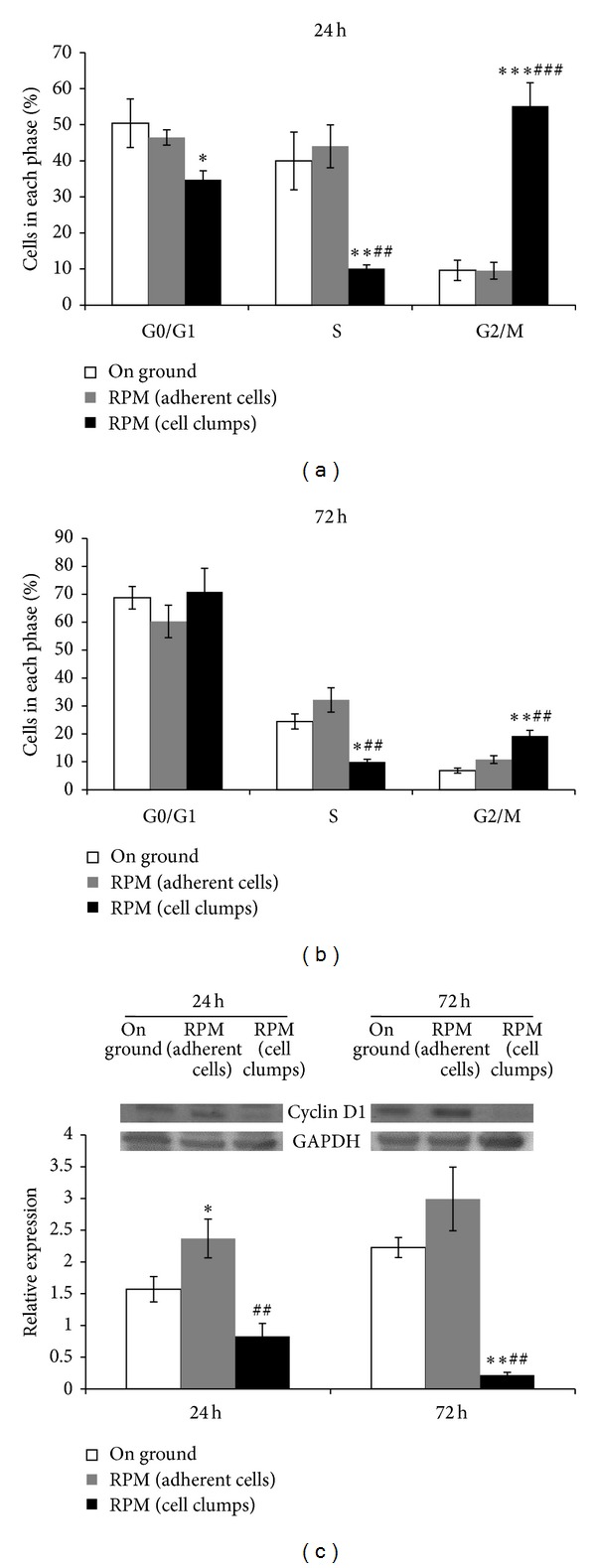
Cell cycle analysis in MDA-MB-231. Cells distribution along the different phases of the cell cycle at 24 (a) and 72 hours (b), in on ground control cells, RPM adherent cells, and RPM cell clumps. (c) Immunoblot bar chart showing the expression of Cyclin D1 in MDA-MB-231 in on ground control cells, RPM adherent cells, and RPM cell clumps at 24 and 72 hours. Columns and bars represent densitometric quantification of optical density (OD) of specific protein signal normalized with the OD values of the GAPDH served as loading control. Each column represents the mean value ± SD of three independent experiments. *P < 0,05; **P < 0,01; ***P < 0,001* versus* on ground control; ^##^ *P* < 0,01; ^###^ *P* < 0,001* versus* RPM adherent cells by ANOVA followed by Bonferroni post-test.

MDA-MB-231 cells subjected to microgravity displayed relevant modification in their cell cycle (Figures 4(a) and 4(b)). Nonadherent RPM-treated MDA-MB-231 cells were distributed in a significantly different manner when compared to both control and RPM-adherent cells; indeed, after 24 hours, floating cell clumps in G0/G1 and in S phase were significantly decreased, whereas cells in G2/M phase increase up to 6-fold. On the contrary, adherent RPM-treated cells displayed only a slight increase in the S phase distribution, when compared to controls. After 72 hours of microgravity exposure, MDA-MB-231 RPM cell clumps still showed a relevant decrease in the S phase, thus demonstrating a persistent inhibition of cell growth; cells number in G2/M phase was significantly higher; meanwhile no significant change in G0/G1 was observed (Figure 4(b)). Again, besides minor differences, control and adherent RPM-treated cells displayed an overlapping distribution in the G0/G1 and G2/M phase, whereas the percentage of cells in the S phase was still higher than that recorded in floating RPM cell clumps. These data are exemplarily mirrored by Cyclin D1 data. Cyclin D1 is one of the main factors that regulate the activation of the cell cycle and its increase is required to foster cell growth. As expected, a statistically significant decrease of Cyclin D1 levels in RPM cell clumps was recorded; meanwhile Cyclin D1 levels are higher in adherent proliferating RPM cells, as well as in control samples. These effects were observed at both 24 and 72 hours (Figure 4(c)).

### 3.5. Microgravity Induces Apoptosis in MDA-MB-231 Cell Clumps

**Figure 5:**
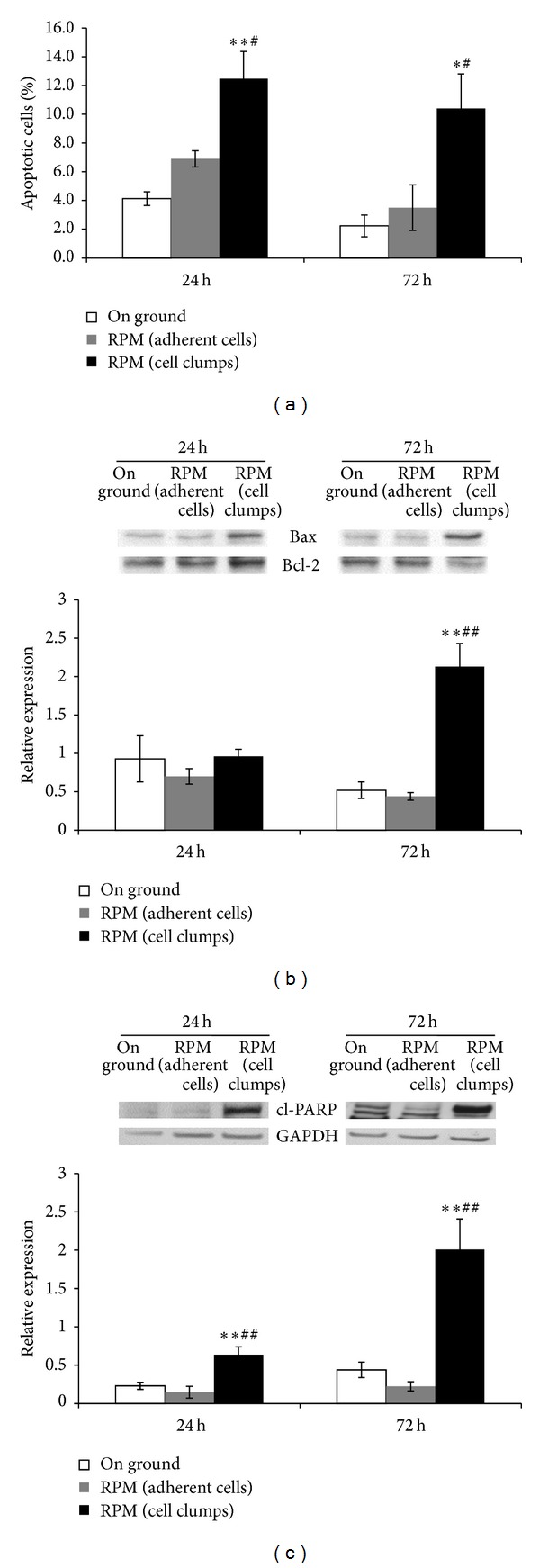
Apoptosis analysis in MDA-MB-231. Apoptotic rate in RPM cultured MDA-MB-231 and on ground cells was determined by a dual parameter flow cytometric assay (a). Histograms show the percentage of apoptotic cells (Annexin V+/7-AAD-); each column represents the mean value ± SD of three independent experiments. Immunoblot bar chart showing the expression of Bax/Bcl-2 ratio (b) and cleaved PARP (c) in on ground control cells, RPM adherent cells, and RPM cell clumps at 24 and 72 hours. Columns and bars represent densitometric quantification of optical density (OD) of specific protein signal normalized with the OD values of the GAPDH served as loading control. Each column represents the mean value ± SD of three independent experiments. *P < 0,05; **P < 0,01* versus* on ground control; ^#^ *P* < 0,05; ^##^ *P* < 0,01* versus* RPM adherent cells by ANOVA followed by Bonferroni post-test.

Data obtained by cytofluorimetric assays reported a statistically significant increase in the apoptotic rate after 24 and 72 hours of microgravity exposure in cell clumps with respect to both adherent cells and on ground control cells (Figure 5(a)). Western blot analysis revealed a statistically significant increase of Bax/Bcl-2 ratio at 72 hours in RPM cell clumps compared to RPM adherent cells and on ground control cells (Figure 5(b)). Similarly cleaved-PARP levels, a direct marker of caspase-3 activation [24], were significantly increased at 24 and 72 hours in RPM cell clumps compared to RPM adherent cells and on ground control cells (Figure 5(c)). Overall these data suggest that nonadherent cells were significantly constrained in their viability, given that microgravity inhibits cell growth and, at the same time, enhances the apoptotic process. Adherent cells in microgravity, on the contrary, display only minor changes in both apoptosis and proliferation rate.

### 3.6. Microgravity Modifies MDA-MB-231 Survival Pathways

**Figure 6:**
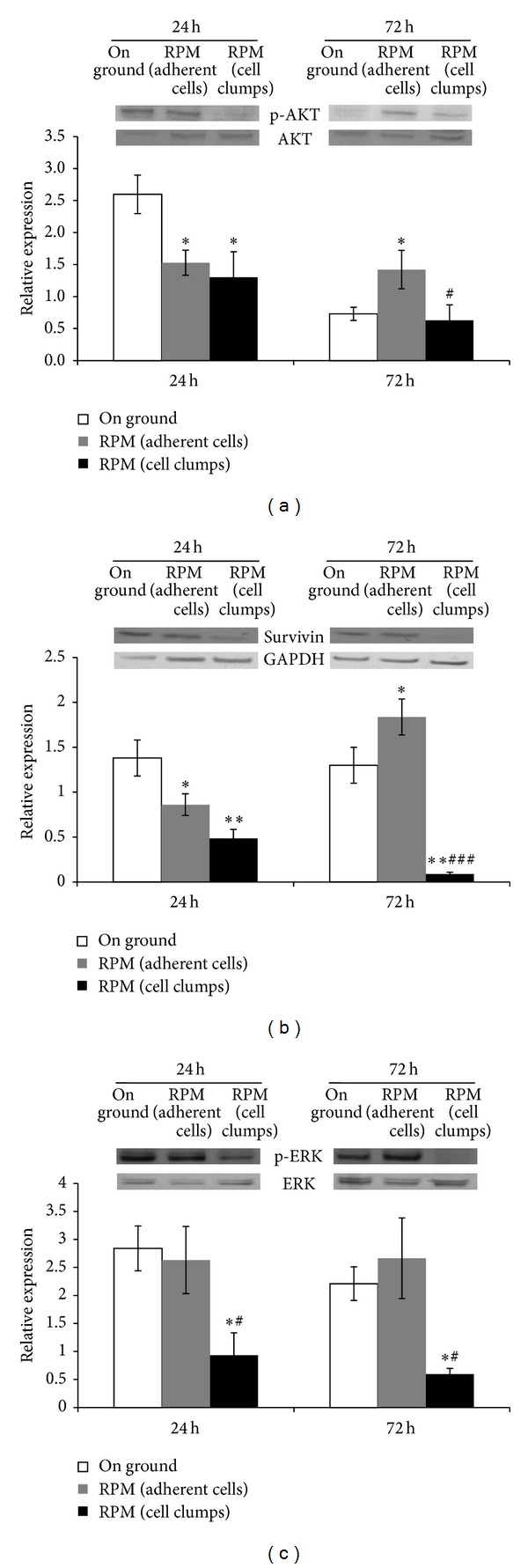
Survival pathways analysis in MDA-MB-231. Immunoblot bar chart showing the expression of p-AKT/AKT ratio (a), Survivin (b), and p-ERK/ERK ratio (c) in on ground control cells, RPM adherent cells, and RPM cell clumps at 24 and 72 hours. Columns and bars represent densitometric quantification of optical density (OD) of specific protein signal normalized with the OD values of the GAPDH served as loading control. Each column represents the mean value ± SD of three independent experiments. *P < 0,05; **P < 0,01* versus* on ground control; ^#^ *P* < 0,05; ^###^ *P* < 0,001* versus* RPM adherent cells by ANOVA followed by Bonferroni post-test.

Microgravity exposure is associated with a statistically significant decrease in the phosphorylation of AKT in adherent cells and cell clumps with respect to on ground control cells after 24 h. Instead, after 72 hours of microgravity exposition, RPM adherent cells showed a statistical increase of p-AKT expression with respect to on ground control cells and RPM cell clumps (Figure 6(a)); such biphasic effect on AKT activation may help explain the biphasic trend observed in apoptosis rate in adherent RPM-exposed cells: apoptosis increases, indeed, at 24 hours when p-AKT values are low; the opposite is observed when p-AKT levels increase at 72 hours. A similar behavior may be described for the two other prosurvival factors, Survivin and phosphorylated-ERK. Microgravity exposure induced a statistically significant decrease in Survivin levels in both adherent and nonadherent RPM-treated cells at 24 hours. However, at 72 hours Survivin levels significantly increased in RPM adherent cells and decreased in nonadherent RPM-treated cells (Figure 6(b)). Likewise, ERK phosphorylation was severely inhibited in RPM cell clumps after 24 and 72 hours, in respect to values observed in both RPM adherent and control cells (Figure 6(c)). Taken as a whole, prosurvival factors increased in adherent RPM-treated cells; meanwhile they decreased in nonadherent RPM-exposed cells, mirroring so far the observed mentioned trend in apoptosis.

## 4. Discussion

Breast cancer cells exposed to microgravity acquire two distinct phenotypes already after the first 24 hours. Such outstanding result has been previously observed in osteoblasts cultured in microgravity [17] and can be interpreted in the light of the nonequilibrium theory. Briefly, a dissipative, nonlinear system sufficiently far from the equilibrium can form spatial stationary patterns after experiencing a phase transition, leading to new asymmetric configurations [25]. These states are equally accessible, that is to say, that there exists a complete symmetry between the emerging configurations, as it is reflected in the symmetry of the bifurcation diagram. However, the superimposition of an external field, even if a weak one like gravity, may break the system's symmetry, bestowing a preferential directionality according to which the system will preferentially evolve into one of the states and not the others. Indeed, bifurcations far from equilibrium endow a system with a very pronounced sensitivity, allowing it to capture the slightest environmental asymmetry and select a preferred polarity or chirality. In other words, the “weak” force dramatically influences the system in selecting one out of many other configurations [26]. On the contrary, by removing the gravitational constraints the system can freely access different attractor states, recovering henceforth new configuration states (“phenotypes”). Such model has been experimentally confirmed, showing that several cell components characterized by a nonlinear dynamics when exposed to microgravity may experience bifurcation transitions, leading to the appearance of new self-organized states from an initially homogeneous conformation [27, 28]. It is tempting to speculate that such transitions may arise in the cell when self-organization processes (cytoskeleton components assembly and mitosis) take place. In our experiment, the annihilation of gravity enables the system to recover more degree of freedom through subsequent symmetry breakings, with the appearance of new morphological and functional phenotypes.

Indeed, MDA-MB-231 cells exposed to microgravity were almost equally split into two distinct populations, characterized by very different morphologies. The first cluster is represented by cells adherent to the substrate, roughly preserving their native, spindle profile. The second one is represented by rounded, smallest cells, grouped and linked to each other, forming aggregates floating in the supernatant.

Fractal analysis provides a quantitative assessment of those qualitative differences [17, 29]. Adherent cells in microgravity showed fractal values significantly higher than suspended cells; coherently, roundness values were greater in suspended than in adherent cells. Additionally, solidity estimation evidences how different these populations are in terms of “potential” deformability. Solidity is a good descriptor of cell deformability, indeed, as it describes in geometrical terms the stiffness and deformability of an object. Thus, the higher the solidity is, the lower the cell deformability is. Nonadherent cells growing in microgravity are grouped in discrete clusters, and they establish tight cell-to-cell contacts. As expected, their solidity value is higher than that recorded in isolated, adherent cells growing in RPM, given that the multiple cell-to-cell adhesion is thought to “stabilize” the cells shape, by mutually reinforcing their stiffness. The combined estimation of these parameters suggests that the two emerging populations significantly exhibit differences in their respective morphological features.

Aggregates of floating cells retain their viability potential and, after reseeding into a normal gravitational field, they are able to fully recover their native morphological traits, already after 24 hours. This is not really an unexpected event, since it has been previously reported that microgravity exposed cells may recover their original profile when replaced in a normal gravity environment [30]. Thereby, gravity-related phenotypic variability may be considered an adaptive, reversible phenomenon.

Changes in cell shape are likely mediated by associated modification in cytoskeleton architecture, which also conveys mechanical stress to the cell biochemical/genetic machinery. Therefore, different cytoskeleton arrangements will end up in activating different gene sequences, leading hence to triggering different biochemical pathways. The balance between tensional forces and the cytoskeleton architecture modulates thereupon several complex cell functions like apoptosis, differentiation, proliferation, ECM remodelling, and so forth [31]. Compelling data demonstrated that both simulated and real, space-based microgravity can severely affect cytoskeleton structure and function [8, 32]. The most impressive modifications were observed in nonadherent RPM-exposed cells in which stress fibers disappear and actin architecture is severely compromised, thus jeopardizing the chances of cell anchoring to the substrate. In the same cells, tubulin microfilaments are almost completely disorganized. This finding may help in explaining the cell cycle inhibition observed in floating cell clumps, given that a correct arrangement of the tubulin meshwork is required to ensure a proper functioning of the mitotic process: microtubules perform indeed a special task during mitosis and meiosis by forming the spindle assembly to align and separate the chromosomes [33]. Yet it is worth of noting that cytoskeleton changes greatly differ between the two RPM-cultured populations, outlining therefore that microgravity enacted the emergence of two very different cytoskeleton phenotypes.

That architectural diversity is associated with significant differences in cell cycle and apoptosis. Adherent breast cancer cells growing in RPM are trying to counteract microgravity effects by increasing the number of cells in the S phase and by stabilizing the apoptotic rate. On the contrary, suspended cell aggregates display a very different behavior, characterized by reduced proliferative capability and enhanced apoptosis.

However, most of the cells in the floating clumps resulted to be viable; in fact, these cells readhered and grew up when once they were reseeded in normal gravity environment. Hence, cell population blocked in G2/M underwent apoptosis; meanwhile cell population blocked in G0/G1 recovered the original profile, when they were reseeded.

It is worth noting that such results have been obtained by treating highly malignant growing cancer cells. In this regard, both cell phenotypes cultured in RPM greatly differ from their counterpart growing in 1 g gravity field. Such processes are remarkably mirrored by the concomitant, coherent changes in several biochemical pathways, mechanistically linked to both proliferation and programmed cell death. Cyclin D1, a key regulatory factor for cell cycle G1/S transition, is significantly increased in adherent MDA-MB-231 cells; meanwhile in suspended cell aggregates Cyclin D1 release is almost completely abolished. Likewise, proapoptotic effectors (BAX, PARP) dramatically increase in suspended RPM-cultured cells; meanwhile prosurvival factors (Bcl-2, Survivin) significantly decrease; Survivin, a well-known critical factor triggering a plethora of survival signaling cascades, was indeed dramatically downregulated and resulted to be undetectable after 72 hours of exposition. Opposite findings were observed in adherent breast cancer cells exposed to microgravity: the Bcl-2 inhibitor of caspase activation increases, whereas proapoptotic effectors concomitantly decline.

Regulation of apoptotic processes relies on the modulation of an intricate interplay between several upstream molecular pathways, involving mainly activation of p-ERK and p-AKT expression. As expected, p-AKT and p-ERK were significantly reduced in suspended cell aggregates; meanwhile they increase in adherent, apoptosis-resistant cells. Overall, these results become evident already at early times, that is, after 24 hours of exposition.

## 5. Conclusions

Our results confirm previous findings, demonstrating that microgravity enacted the emergence of distinct phenotypes, characterized by significant, recognizable differences in shape configuration, biochemical pathways architecture, and behavioral processes [17]. Additionally, it should be remarked that the coexistence of two different cell populations may contribute to explain some contradictory results provided by earlier studies [34, 35]; indeed, increase or reduction in cell proliferation as well as enhanced or reduced apoptosis could well be both found during microgravity experiments, given that such opposite behaviors must be ascribed to very different cell clusters.

Spontaneous emergence of different phenotypes in microgravity after the system has experienced a symmetry breaking is a finding worth of interest and may have relevant consequences for human space flights. Phenotypic switch leading to divergent morphological and biochemical configuration is triggered by nonlinear processes taking place near the transition point. Such transition enables the system to recover new degree of freedom and, as such, it may be viewed as a spontaneous process allowed by the nonequilibrium thermodynamics. That finding highlights the relevance of biophysical constraints in shaping the form biological, dissipative systems acquire [36] and may help understand how cells and tissues behave during development, pathological events, or in extreme environmental fields.

## Figures and Tables

**Table 1:** 

	Roundness	±SE		Solidity	±SE		FD	±SE	
24 hours									
On ground cells	0,4563	0,0301		0,6690	0,0225		1,7482	0,0091	
RPM adherent cells	0,3991	0,0275		0,6499	0,0200		1,7406	0,0063	
RPM cell clumps	0,7894	0,0219	∗∗	0,8966	0,0263	∗∗	1,4625	0,0015	∗∗
72 hours									
On ground cells	0,3227	0,0263		0,4687	0,0136		1,6677	0,0036	
RPM adherent cells	0,4081	0,0311		0,6115	0,0226	∗	1,7245	0,0067	
RPM cell clumps	0,7961	0,0208	∗∗	0,8573	0,0469	∗∗	1,4990	0,0015	∗∗

Roundness, solidity, and fractal dimension (FD) mean values ± SE in on ground control cells, RPM adherent cells, and RPM cell clumps. ∗*P* < 0.01 *versus* on ground control cells; ∗∗*P* < 0.001 *versus* on ground control and RPM adherent cells by ANOVA followed by Bonferroni post-test.
